# The Universal Neighborhood Effect Averaging in Mobility-Dependent Environmental Exposures

**DOI:** 10.1021/acs.est.4c02464

**Published:** 2024-10-03

**Authors:** Jiannan Cai, Mei-Po Kwan

**Affiliations:** † Institute of Space and Earth Information Science, The Chinese University of Hong Kong, Shatin, Hong Kong, China; ‡ Department of Geography and Resource Management, The Chinese University of Hong Kong, Shatin, Hong Kong, China

**Keywords:** environmental exposures, human mobility, neighborhood effect averaging problem, spatial autocorrelation, multiscenario simulation

## Abstract

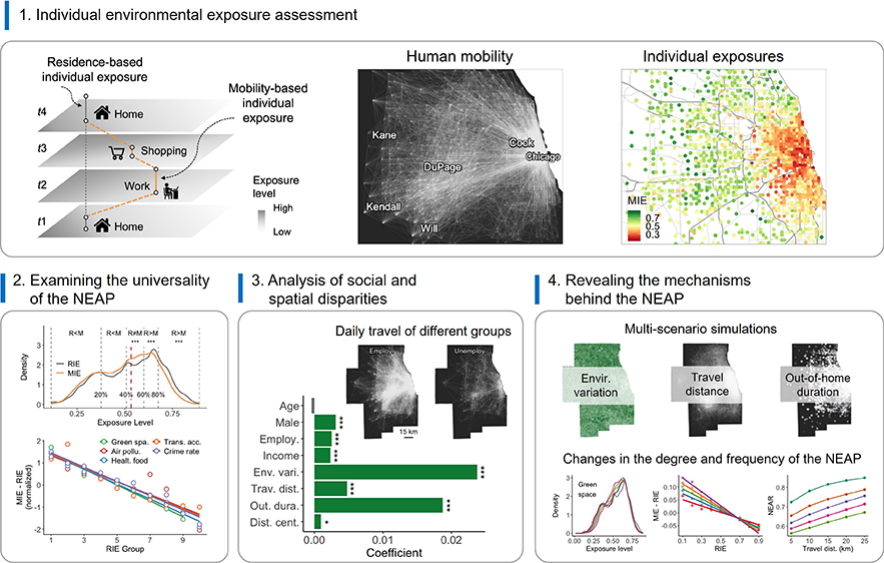

The neighborhood effect averaging problem (NEAP) is a fundamental statistical phenomenon in mobility-dependent environmental exposures. It suggests that individual environmental exposures tend toward the average exposure in the study area when considering human mobility. However, the universality of the NEAP across various environmental exposures and the mechanisms underlying its occurrence remain unclear. Here, using a large human mobility data set of more than 27 000 individuals in the Chicago Metropolitan Area, we provide robust evidence of the existence of the NEAP in a range of individual environmental exposures, including green spaces, air pollution, healthy food environments, transit accessibility, and crime rates. We also unveil the social and spatial disparities in the NEAP’s influence on individual environmental exposure estimates. To further reveal the mechanisms behind the NEAP, we perform multiscenario analyses based on environmental variation and human mobility simulations. The results reveal that the NEAP is a statistical phenomenon of regression to the mean (RTM) under the constraints of spatial autocorrelation in environmental data. Increasing travel distances and out-of-home durations can intensify and promote the NEAP’s impact, particularly for highly dynamic environmental factors like air pollution. These findings illuminate the complex interplay between human mobility and environmental factors, guiding more effective public health interventions.

## Abstract 2

Ignoring people’s daily mobility could bias the estimation of individual environmental exposures. This study analyzes the sources, manifestations, and generation of biases in individual exposure estimates, informing better public health interventions.

## 1 Introduction

As cities undergo multifaceted socioecological transformations, individuals become intricately woven into a rich tapestry of diverse natural, built, and social environments on a daily basis. To better inform urban planning and environmental policy interventions, there is an imperative need to quantitatively assess the daily environmental exposures experienced by individuals. Past research mostly employed individuals’ residential units, often operationalized as static geographic areas, as the contextual units for deriving environmental exposure estimates. This approach is rooted in the assumption that people’s health outcomes are primarily shaped by their residential environments, such as green spaces or air quality in their residential units.^1,2^ However, individuals commonly move from place to place during the day for routine activities like work, study, socializing, and recreation, resulting in dynamic exposure to a wider range of environmental conditions beyond their residential units. Disregarding people’s daily mobility could lead to inconsistent and even unreliable estimations of environmental exposures and their associated health effects.^3,4^ In light of this, recent research has been integrating contextual information with human mobility patterns to more accurately estimate individuals’ environmental experiences in their actual mobile settings, such as air pollution exposures,^5−7^ social segregation experiences,^8−12^ and flood risk exposures.^13^

The increasing body of scientific evidence on the high interpretability and predictability of individual mobility patterns^14−17^ suggests that the environmental exposures individuals experience during their movements may harbor explicable or predictive regularities. In particular, it has been observed that individual exposures to environmental factors tend to regress toward the average exposure value of the population or participants in the study area when accounting for people’s daily mobility. This statistical phenomenon is named the neighborhood effect averaging problem (NEAP).^18^ It can, to some extent, be explained by the well-known statistical phenomenon of regression to the mean (RTM),^19,20^ which refers to the tendency of extreme measurements to move closer to the population mean upon repeated measurements due to natural random variations without any intervention. The RTM has been widely observed in various aspects of everyday life, such as changes in offspring’s height,^21^ biomarker measurements,^22^ and more recently, variations in commute times after job or home moves.^23^ Similarly, extreme environmental exposures experienced in fixed residential units can be moderated through dynamic exposure to a wider range of environments as individuals move through diverse units during their daily mobility. In other words, individuals residing in residential units with higher (or lower) exposure to the environmental factor of interest are more likely to experience lower (or higher) exposure values when they visit units outside their homes. As a result, individual exposure values during daily mobility tend to regress more toward the average exposure value compared to those experienced in fixed residential units, though the degree of regression may vary depending on individual mobility patterns. Therefore, assessing individual exposures based on the environmental factors only in people’s residential units may lead to unreliable and erroneous conclusions regarding mobility-dependent exposures and their health impacts. Recently, some studies have provided preliminary evidence for the existence of the NEAP in several kinds of individual exposures, such as individual air pollution exposures^24^ and ethnic segregation.^25^ However, it remains unclear whether the NEAP is universally present in other mobility-dependent exposures, such as green spaces and healthy food environments, because different environmental factors could have varying spatiotemporal dynamics and interactions with human mobility patterns. Furthermore, the underlying mechanisms giving rise to the NEAP in individual environmental exposures are not yet fully understood.

Therefore, in this study, we examine the universality of the NEAP in individual exposures to diverse mobility-dependent environmental factors, including green spaces, air pollution, healthy food environments, transit accessibility, and crime rates, using large-scale human mobility data in the Chicago Metropolitan Area. Further, we identify which social groups and residential areas are more susceptible to the influence of the NEAP on the estimation of individual environmental exposures. Moreover, we address how environmental variation and individual mobility characteristics contribute to the occurrence of the NEAP through a substantial number of multiscenario simulations. This endeavor can provide crucial theoretical and statistical foundations for understanding the sources and generation of uncertainties in individual exposure assessments involving all kinds of mobility-dependent environmental factors. Further, our research findings can inform evidence-based decision-making and contribute to the development of more effective and impactful policies in public health and urban planning.

## 2 Materials and Methods

### 2.1 Data

#### 2.1.1 Human Mobility Data

Our human mobility data comes from a large-scale daily travel survey conducted by the Chicago Metropolitan Agency for Planning (CMAP) between August 2018 and April 2019. The survey area covers nine counties in the Chicago Metropolitan Area: Cook, DeKalb, DuPage, Grundy, Kane, Kendall, Lake, McHenry, and Will. In that survey, participants provided personal socio-demographic information, such as age, gender, employment status, and household income. In addition, they were asked to report the details of daily trips, including origins, destinations, start and end times, and travel modes, for a designated weekday. The original travel survey includes 28 577 participants. After excluding participants with incomplete essential information or those who moved outside the survey area, a total of 93 333 daily trips from 27 198 individuals are considered in this study. The travel paths between each trip’s origin and destination are inferred using the fastest travel route corresponding to the reported travel mode^26^ (see Text S1). To facilitate integration with environmental data at various spatial resolutions, all spatial locations are further aggregated into 1 km × 1 km grid cells. This resolution is widely adopted for analyzing large-scale human mobility patterns^17,27^ and is well-suited for capturing individual movements in this study, given participants’ average daily travel distance of 28.3 km.

#### 2.1.2 Environmental Data

We collect data on various natural, built, and social environmental factors, including green spaces, air pollution, healthy food environments, transit accessibility, and crime rates. These environmental factors are characterized using the Normalized Difference Vegetation Index (NDVI), hourly Particulate Matter 2.5 (PM_2.5_) concentration, the modified retail food environment index (mRFEI),^28^ proximity to transit stops,^29^ and the number of violent crimes per 1 000 residents, respectively. Data on green spaces, air pollution, and healthy food environments cover the nine-county area, whereas transit accessibility and crime rates are limited to seven counties and the City of Chicago, respectively, due to limitations in data availability. Despite temporal differences in the data, we assume that the travel survey can adequately represent the routine daily mobility of individuals, thus making it feasible to investigate the NEAP in individual environmental exposures. We aggregate all environmental data into 1 km × 1 km grid cells for easy integration with individual mobility data. This resolution strikes a balance between capturing spatial variation and ensuring computational feasibility. This is particularly crucial for our extensive multiscenario analyses (Section 2.5), as the computational complexity heavily depends on the observed resolution, making computations impractical at finer resolutions. Sensitivity testing further demonstrates that this resolution can provide evidence concerning the NEAP that is statistically consistent with that obtained with finer resolutions (Figure S7). Details about the availability, collection, processing, estimation and visualization of these environmental data can be found in Texts S2, S3, and S9 and Figures S4 and S6.

### 2.2 Measuring Individual Environmental Exposures

**Figure 1:**
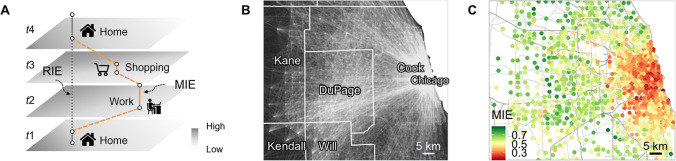
Individual environmental exposures. (A) Schematic illustration of RIE and MIE. (B) Individual daily travel flows in the Chicago Metropolitan Area. A zoomed-in map is given to facilitate a better interpretation. (C) MIE to green space. For privacy protection considerations, the displayed locations are the centers of 1 km × 1 km grid cells where each individual’s home is located.

To measure the level of environmental exposure experienced by individuals during their daily mobility, we further link environmental factors to the geographic context of individual mobility patterns (Figure 1A). For each individual *i*, we retrieve his or her grid location at each minute *t* and obtain the level of the studied environmental factor at that specific location and moment, IE_*i,t*_. Then, the mobility-based individual environmental exposure MIE_i_ is defined as the average level of environmental exposures experienced by individual *i* across various units visited at different time points throughout the day:
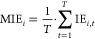
1where *T* is the total time in a day and equals 1 440 min. In contrast, the residence-based environmental exposure of individual *i*, RIE_*i*_, is measured as the daily average level of the studied environmental factor at the residential grid location by assuming individual *i* always stays at his or her home.

### 2.3 Quantifying the NEAP in Individual Exposures

The estimation of MIE considers not only the residential exposure shared by individuals living in the same unit but also the nonresidential exposures encountered in other visited units, which are more akin to the experiences of a broader range of individuals. This is because daily mobility offers individuals more potential opportunities for richer social interactions and exposure to different levels of environmental factors. As a result, compared to the RIE, the MIE is more likely to regress toward the average exposure level of the population or participants in the study area, thereby undergoing neighborhood effect averaging.^18^ We observe the manifestations of the NEAP from two perspectives: (1) the extent to which individual environmental exposure estimates are influenced by the NEAP and (2) the proportion of individuals whose environmental exposure estimates are impacted by the NEAP. Question (1) is addressed through the degree of negative correlation between MIE-RIE and RIE. To address (2), we introduce a metric, the Neighborhood Effect Averaging Ratio (NEAR), to determine the frequency of individuals situated in specific tails of the RIE distribution on both ends being impacted by the NEAP. The tails of the RIE distribution are crucial for understanding the frequency of the averaging process of individual exposures because individuals with more extreme RIE levels are more likely to exhibit significant changes during daily mobility. An increase in MIE for individuals with extremely low RIE and a decrease for those with extremely high RIE both indicate a regression toward the average exposure, representing upward and downward averaging, respectively. By focusing on these RIE extremes, the NEAR tracks how many of these individuals’ MIE levels experience changes toward the average, thus providing a clear metric to quantify the frequency of the NEAP’s effect on individual exposure estimates. Specifically, for the bottom and top α% tails of the RIE distribution,  and , of all participants in the study area, the NEAR is calculated as
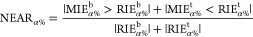
2where  represents the number of individuals within the bottom α% tail of the RIE distribution whose MIE exceeds their RIE, and  is analogous but for the top *a* % tail of the RIE distribution. The NEAR_α%_ is bounded between 0 and 1. A higher value suggests that more extreme residential exposures are moderated through daily mobility, implying that a greater number of individuals experience the NEAP in their daily exposures.

### 2.4 Modeling the Impact of the NEAP

Although the above metrics can examine the presence of the NEAP, the impact of the NEAP on individual exposure estimates may vary both among individuals and across space. This is because individuals from different social groups and residential units may have distinct daily mobility patterns and are situated in different environmental contexts. We further model the impact of the NEAP on individual exposure estimates at the individual level using a spatial error model. The extent to which individual exposure estimate is influenced by the NEAP is measured based on the difference between RIE and MIE, i.e., . We consider four dimensions that could affect the extent of the NEAP’s impact: (1) individual socio-demographic attributes, including age, gender, employment status and household income; (2) residential location, i.e., the distance between one’s home and the primary city center within the county; (3) environmental variation, i.e., the variance of the environmental factor under investigation within the individual’s daily activity space defined based on his or her trajectories; and (4) human mobility, including the total travel distance and duration of stay at out-of-home locations during the survey day. Travel time is not considered here to avoid collinearity with travel distance. In addition, to compensate for the difference between areas in the Chicago Metropolitan Area, we also include geographical fixed effects at the county level. Dimensions (1) and (2) are used to explain the social and spatial disparities in the NEAP’s impact on individual exposure estimates. Dimensions (3) and (4) can reveal how individuals’ environmental contexts and mobility patterns shape the NEAP’s impact on their exposure estimates.

### 2.5 Revealing the Mechanisms behind the NEAP

Real-world sampling data can empirically demonstrate the existence of the NEAP and its impact on individual exposure estimates but fall short in deeply analyzing the mechanisms underlying its occurrence, specifically the degree and frequency of the NEAP in relation to two triggering factors: environmental variation and human mobility. This is because the scenarios of environmental variation and human mobility captured by sampled data are limited and potentially influenced by multiple complex factors, making independent control challenging. Therefore, we perform multiscenario analyses based on simulations of environmental variation and human mobility. Through simulations, we can independently control the levels of environmental variation and human mobility, eliminating the influence of other confounding factors. Then, the patterns of the degree and frequency of the NEAP changes with environmental variation and human mobility are determined by varying one factor through simulations while keeping the other constant as observed in the real-world data set. This allows us to more deeply study the mechanisms by which these two triggering factors affect the NEAP. Specifically, we characterize environmental variation from its opposite perspective, spatial autocorrelation, using a well-established spatial statistic, Moran’s *I* (*MI*).^30^ The spatial resolution and value set of the simulated environmental data are identical to those of the observed NDVI data; however, the observed values are spatially permuted across grid cells to achieve the desired level of spatial autocorrelation using a modified spatial autocorrelation reconstruction method^31^ (Text S4). Similarly, we characterize human mobility based on individuals’ daily travel distance and out-of-home duration, and then control them to specified levels through simulations, while keeping the number of individuals and their residential locations identical to those in the observed data set (Text S5). To achieve statistically robust results, each scenario of environmental variation or human mobility is simulated multiple times (999 times in this study).

## 3 Results

### 3.1 The NEAP in Individual Environmental Exposures

As shown in Figure 1B, a large portion of residents living in different communities in the Chicago Metropolitan Area commonly travel to areas in the urban core (such as downtown Chicago) during the day for various purposes like work, shopping, and more. The daily mobility of individuals leads to more dynamic environmental exposures, making it unrealistic to use people’s residential units as their environmental contexts. Figure 1C displays the spatial distribution (by home location) of individual exposure levels to green spaces in the Chicago Metropolitan Area, taking into account people’s daily mobility. It shows that MIE levels are highly heterogeneous across space, and even individuals residing within the same community can exhibit significant differences in their MIE levels. Specifically, the Mann–Whitney U test reveals statistically significant differences between MIE and RIE levels, with a *p*-value less than 0.001. In 49.7% of residential grid cells, the coefficient of variation of participants’ MIE levels exceeds 0.1, reaching a maximum of 0.48. This suggests that individuals, even residing within the same community, can experience markedly different environmental exposures during their daily mobility.

**Figure 2:**
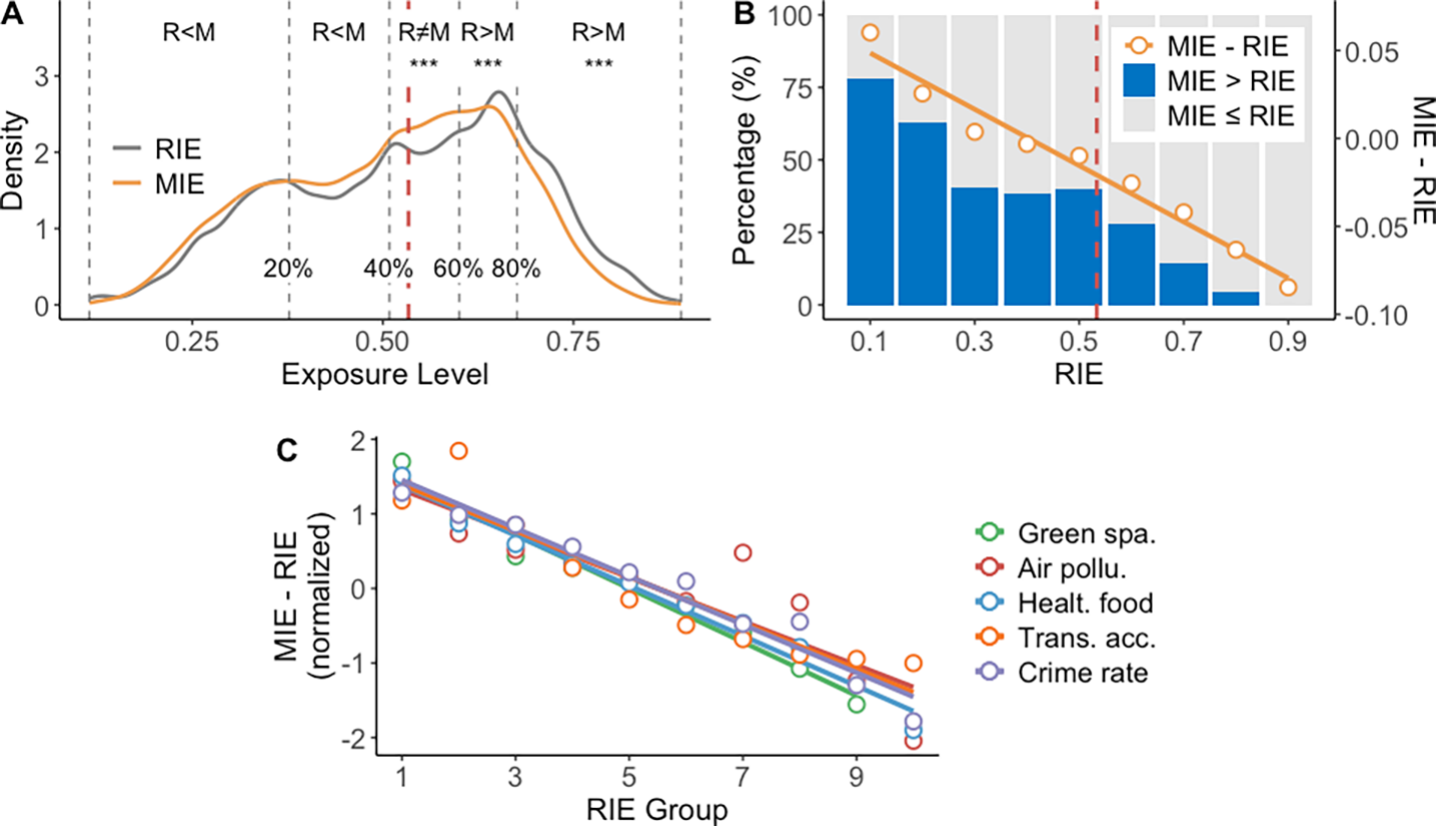
Evidence of the NEAP in individual exposures. (A) Comparison of distributions of RIE and MIE to green space. The red vertical dashed line represents the average value of RIE. *** indicates that the *p*-value of the Mann–Whitney *U* test comparing the RIE within a specific value range to the MIE of the same individuals is less than 0.001. (B) Evidence of the NEAP in individual green space exposure. The percentage of increasing and decreasing MIE for each interval of RIE is displayed. The fitted line demonstrates a significant negative linear correlation between MIE-RIE and RIE. Specifically, MIE-RIE = −0.16 × RIE + 0.06, *R*^2^ = 0.97, *p*-value <0.001. (C) Evidence of the NEAP in individual exposures to various environmental factors. For each environmental factor, the MIE-RIE values are normalized on the *y*-axis, and the RIE values are discretized into ten groups on the *x*-axis. The linear regressions reveal a universally significant negative correlation between MIE-RIE and RIE, with slopes ranging from −0.36 to −0.29, *R*^2^ values from 0.79 to 0.97, and *p*-values all less than 0.001.

Figure 2A compares the probability distributions of MIE and RIE to green spaces. Notably, around the average value of RIE (see the red vertical dashed line), the probability density of MIE is typically higher than that of RIE. This indicates that when considering people’s daily mobility and nonresidential exposures, individuals are more likely to experience the average level of green space exposures of participants in the study area. Further, Figure 2B aggregates the percentage of increasing or decreasing MIE to green spaces for each interval of RIE. We can observe a notable manifestation of neighborhood effect averaging: individuals with lower RIE are more likely to experience higher MIE, while individuals with higher RIE are more likely to experience lower MIE. This phenomenon is further supported by a significant negative linear correlation between the variation of the MIE level and the baseline RIE level. In addition to green space exposure, the widespread negative correlation presented in Figure 2C also provides evidence for the existence of the NEAP in relation to other mobility-dependent environmental factors in our data set, including air pollution, healthy food environment, transit accessibility, and crime rates (see Text S7).

Further, we investigate whether the NEAP always exists over different value ranges of environmental exposure estimates. Figure 2A divides the whole value range of RIE to green spaces into five subranges based on quintiles. To test the trend of MIE regressing toward the average exposure level, we perform Mann–Whitney *U* tests to analyze whether the MIE level of a participant is significantly larger than his/her RIE level for the first and second quintiles of RIE. For the fourth and fifth quintiles, we test whether the MIE level is smaller than the RIE level. For the middle quintile, we are only interested in whether there is a difference between the MIE and RIE levels. Statistically significant Mann–Whitney *U* test results (*p*-value <0.001) can only be observed for the middle, fourth and fifth quintiles, while the first and second quintiles do not show statistical significance. The results suggest that limited samples of individual environmental exposures (e.g., those of individuals from communities with similar residential environments) may not comprehensively reveal the NEAP because they are “short-sighted” and say little about the NEAP over the whole value range of exposure levels of the population or participants in the study area.

### 3.2 Explaining the NEAP

**Figure 3:**
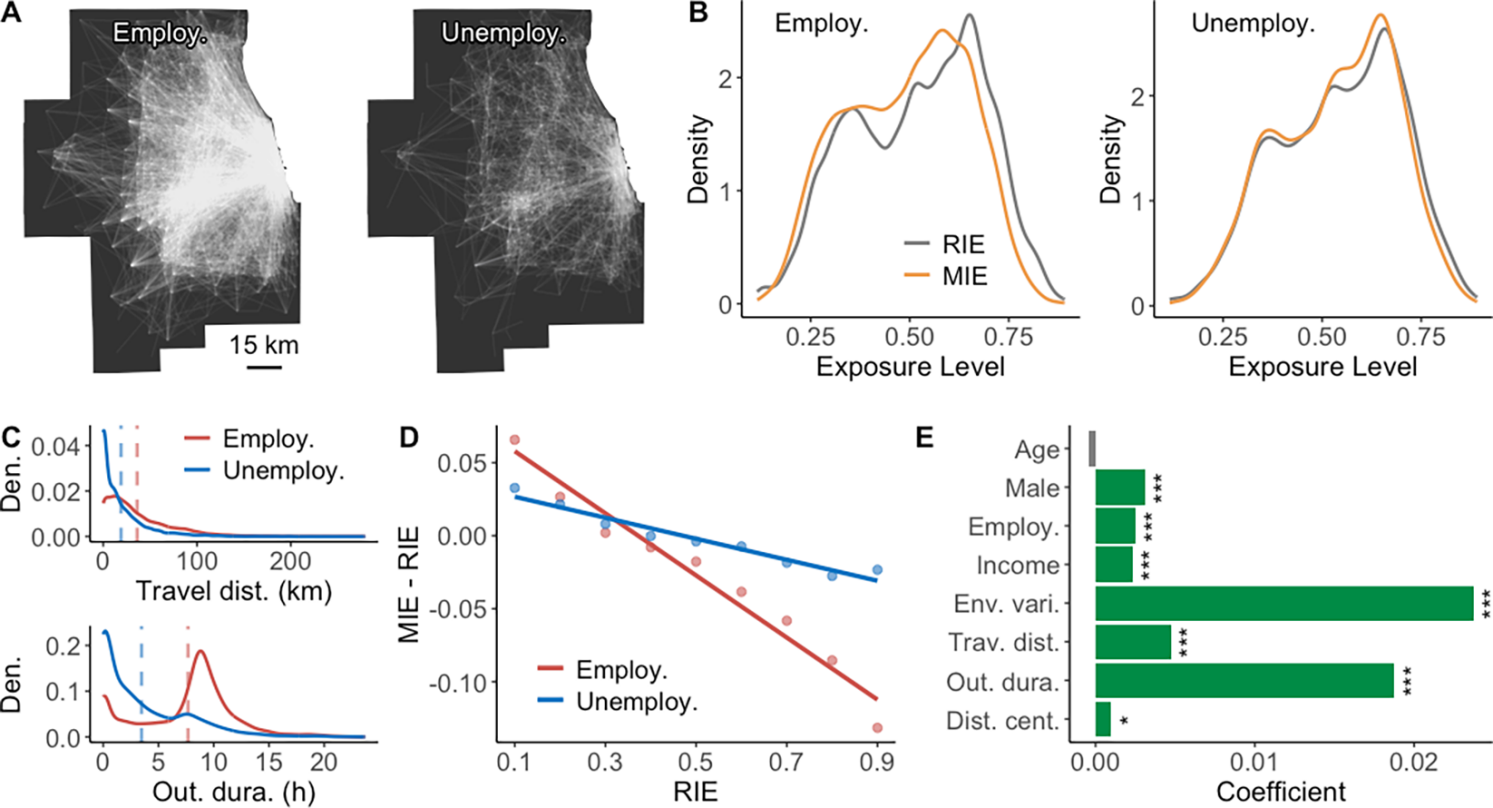
Different social groups experience varying levels of the NEAP. (A) Individual daily travel flows of employed and unemployed groups. (B) Comparison of distributions of RIE and MIE to green space experienced by employed and nonemployed population groups. (C) Comparison of distributions of the travel distance and duration of stay at out-of-home locations between employed and nonemployed groups. The red and blue vertical dashed lines represent the average values of travel statistics of employed and nonemployed groups, respectively. (D) Comparison of the NEAP in individual green space exposure between employed and nonemployed groups, implied by the degree of negative correlation between MIE-RIE and RIE. (E) Summary of coefficients of all variables in the spatial error model for estimation bias in individual exposure (i.e., ). ***p-value <0.001. *0.01*p*-value <0.05.

Figure 3E summarizes the coefficients of all variables in the spatial error model for explaining individual exposure estimation bias, i.e., , in relation to green space in the Chicago Metropolitan Area. It demonstrates that greater environmental variation within individuals’ activity spaces, along with longer daily travel distances and out-of-home durations, can significantly heighten the risk of individual exposure estimates being influenced by the NEAP; see Text S8.

The results also show that individuals who are male, employed and have higher household incomes tend to exhibit larger differences between RIE and MIE concerning green spaces, suggesting a higher impact of the NEAP on individual exposure estimates. However, there is no significant relationship between age and the extent of NEAP’s impact. Take employment status as an example. Employed individuals show more active daily mobility compared to unemployed individuals due to their commuting needs for work-related activities (Figure 3A). Specifically, employed individuals have larger average daily travel distances and longer durations of stay at out-of-home locations (36 km and 7.7 h, respectively), compared to those (19 km and 3.5 h, respectively) of unemployed individuals (Figure 3C). The limited daily mobility of unemployed people makes it difficult for them to significantly increase or decrease their daily exposures, thus their MIE distribution is quite similar to their RIE distribution (Figure 3B). In contrast, employed individuals can encounter a wide range of individual exposures during their daily mobility compared to their residential areas, as they tend to stay longer in places that are located farther from their homes, resulting in potentially greater environmental variation in their activity space. Figure 3D further demonstrates that employed individuals witness a more pronounced NEAP in their individual exposures compared to unemployed individuals.

**Figure 4:**
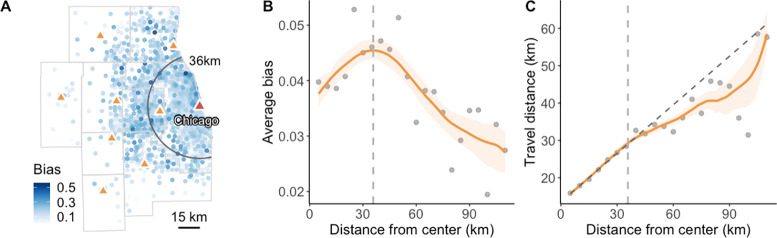
Bias in the estimation of individual environmental exposure as a function of the distance of home locations from the primary city center. (A) The spatial distribution (by home location) of estimation biases in individual green space exposure in the Chicago Metropolitan Area. The red triangle represents the city center of Chicago, while the orange triangles represent city centers in the other eight counties. (B,C) Average bias (B) and travel distance (C) with varying distances from the city center of Chicago. The vertical dashed line represents the critical distance (36 km) from the city center at which the estimation bias begins to decrease. We also apply the nonparametric fitting for each plot and calculate the bootstrap-based 95% confidence interval of each fitting curve. The *R*^2^ values for the nonparametric fits in (B) and (C) are 0.71 and 0.84, respectively. The black dashed line in (C) represents the linear fit of travel distance with respect to distance from the center within the critical distance, with an *R*^2^ of 0.996.

Further, we investigate the spatially heterogeneous effect of the NEAP on individual exposure estimates in different residential locations. Using the distance from each individual’s home location to the city center of Chicago (see the red triangle in Figure 4A) as an indicator, Figure 4B presents an inverted U-shaped curve between the distance and the average bias in the estimation of individual green space exposure. As the residential distance from the city center increases, the average exposure estimation bias initially increases, but it begins to decrease after reaching 36 km. To explain this, Figure 4C summarizes the changes in daily travel distances of participants with respect to their residential distance from the city center of Chicago. Within the critical distance of 36 km, it is evident that the average travel distance of individuals increases linearly with their residential distance from the city center. This results in individuals residing farther from the city center having more opportunities to experience varying degrees of environmental exposure in their daily travels, thus leading to an increase in exposure estimation bias. However, as the residential distance continues to increase, the rate of increase in the average travel distance begins to deviate from the expected linear trend (see the black dashed line in Figure 4C). This suggests that the attractiveness of the primary city center for individual travel has a distance-based upper bound.^27^ In line with spatial economic theory,^32^ individuals located outside this attraction bound may weigh the trade-off between the amenities provided by the city center and the costs associated with travel, potentially opting for closer locations for their daily activities. This, in turn, reduces the variation in environmental exposure during individual daily mobility, leading to a reduction in exposure estimation bias.

Considering the bounded attraction of the city center, we further narrow our focus to different counties to explain the spatially heterogeneous effect of the NEAP. Consistent with the pattern observed within the attraction bound of the city center of Chicago, the distance from an individual’s home to the primary city center within their county has a significant positive effect on individual exposure assessment bias (Figure 3E). This suggests that the NEAP tends to dominate spatially on the city’s outskirts, where residents commonly have longer daily commuting distances, thus amplifying the likelihood of experiencing greater variability in environmental exposures during their daily mobility.

### 3.3 The Influence of Environmental Variation on the NEAP

**Figure 5:**
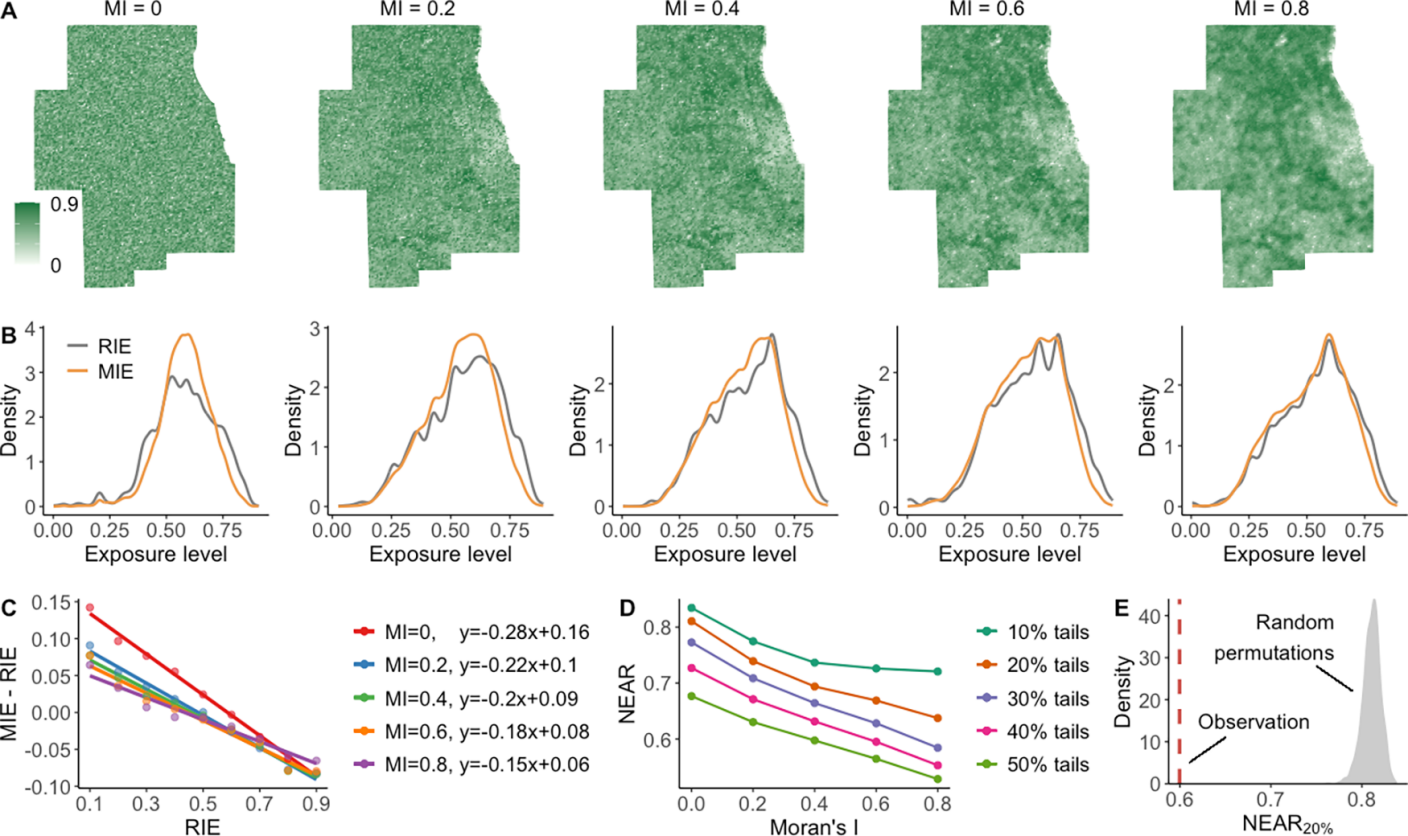
Influence of environmental variation on the NEAP. (A) Example simulated environmental data sets with different spatial autocorrelation levels in the Chicago Metropolitan Area. The simulated values are identical to the observed NDVI, but they are spatially permuted at different autocorrelation levels (MI = 0, 0.2, 0.4, 0.6, 0.8). (B) Comparison of distributions of RIE and MIE experienced in different simulated environmental data sets. (C) Comparison of NEAP in different simulated environmental data sets, implied by the degree of negative correlation between MIE-RIE and RIE. (D) Comparison of the NEAR at different levels of spatial autocorrelation. For each spatial autocorrelation level, the NEAR is measured for the 10%, 20%, 30%, 40%, and 50% tails of the distribution of RIE, and the results are averaged over 999 simulations. (E) Distribution of NEAR for the 20% tails of RIE (NEAR_20%_) under the scenario of complete spatial randomness. The distribution is estimated based on 999 complete spatial randomizations of the observed NDVI. The vertical red dashed line represents the NEAR_20%_ value (0.6) estimated in the observed NDVI data set with an MI value of 0.67.

Figure 5A illustrates example simulated data sets on 1 km × 1 km grid cells in the Chicago Metropolitan Area under five distinct environmental variation scenarios (MI = 0, 0.2, 0.4, 0.6, 0.8). In each simulated data set, values are derived from the observed NDVI, but they are spatially permuted to achieve the desired level of spatial autocorrelation. As we can see, under a scenario of lower environmental variation characterized by a higher MI value, individuals experience more comparable levels of environmental exposure during their daily mobility, leading to a convergence of MIE toward RIE (Figure 5B). Therefore, the increasing MI value can contribute to the weakening of the negative correlation between MIE-RIE and RIE (Figure 5C), implying a diminishing effect of the NEAP on individual exposure estimates. In addition, Figure 5D demonstrates that in scenarios with lower environmental variation, fewer individuals experience the NEAP. The results suggest that, as spatial autocorrelation strengthens, the environmental variation decreases, resulting in a weaker NEAP effect and fewer individuals experiencing it.

Specifically, when the environmental factor is distributed without any spatial constraints, meaning it is completely randomly spread across space (i.e., MI = 0), individual movement in space can result in an equally likely transition of RIE to any level of MIE. For instance, individuals at the lower (or upper) 20% of the RIE have an 80% chance of experiencing an increase (or decrease) in MIE. This is the result of the RTM observed under random variations. However, due to the inherent spatial autocorrelation in environmental data (MI = 0.67 for the NDVI), the NEAR value of individual exposures to the observed NDVI (NEAR_20%_ = 0.6) is significantly lower than those estimated under the scenario of complete spatial randomness (Figure 5E). This explains the difference between the NEAP and RTM: the NEAP is a statistical phenomenon of RTM under the spatial autocorrelation constraints of environmental data. Because the spatial autocorrelation of environmental factors constrains the variation in individuals’ exposure levels during their movements, it diminishes the impact of RTM, resulting in the still evident NEAP, which exhibits a weakened tendency to regress toward the mean.

### 3.4 The Influence of Human Mobility on the NEAP

**Figure 6:**
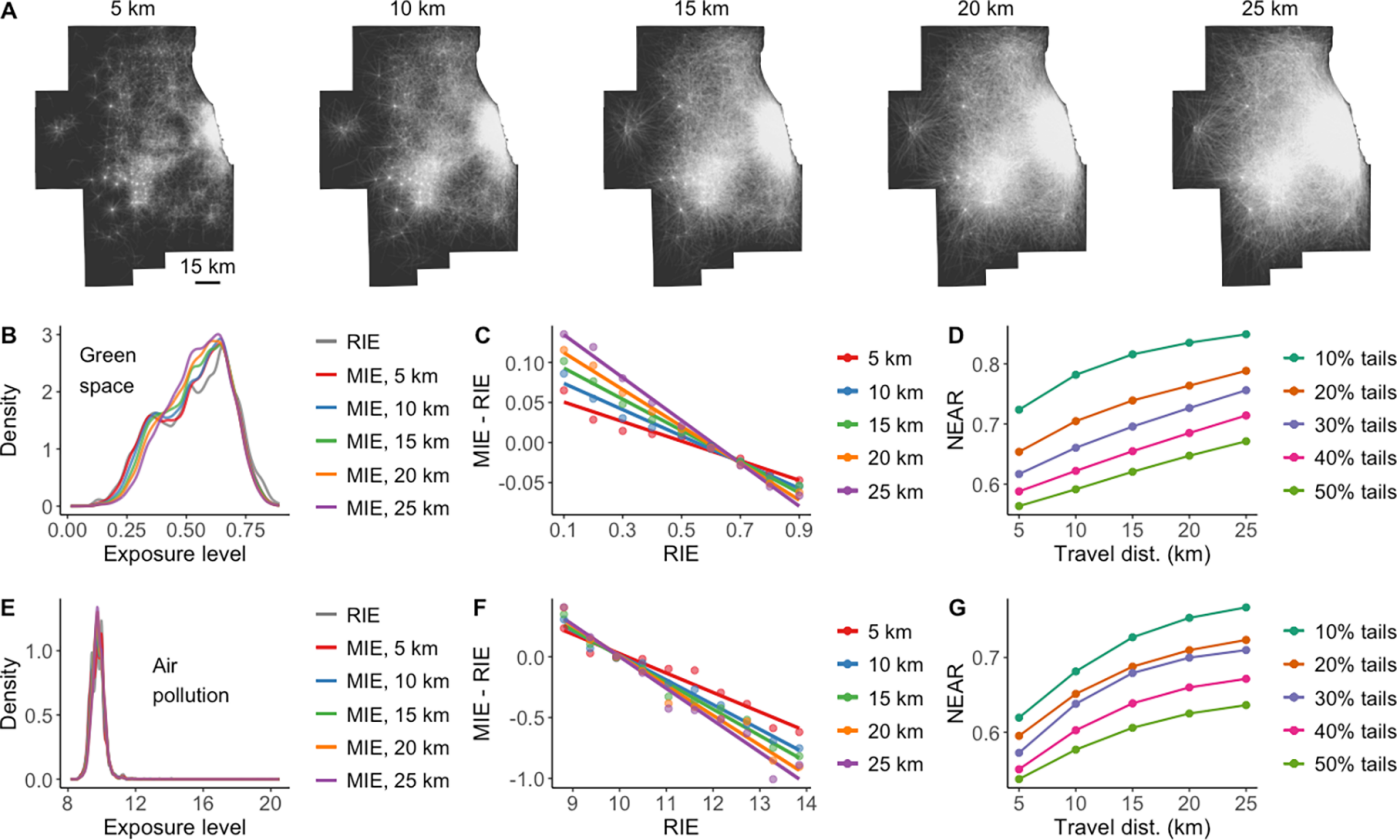
Influence of travel distances on the NEAP. (A) Example simulated mobility data sets with different travel distances in the Chicago Metropolitan Area. (B,E) Comparison of distributions of RIE and MIE to green space (B) and air pollution (E) in simulated data sets with different travel distances. (C,F) Comparison of NEAP in relation to green space (C) and air pollution (F) in simulated data sets with different travel distances, implied by the degree of negative correlation between MIE-RIE and RIE. (D,G) Comparison of the NEAR in relation to green space (D) and air pollution (G) under different travel distances. For each travel distance, the NEAR is measured for the 10%, 20%, 30%, 40%, and 50% tails of the distribution of RIE, and the results are averaged over 999 simulations.

**Figure 7:**
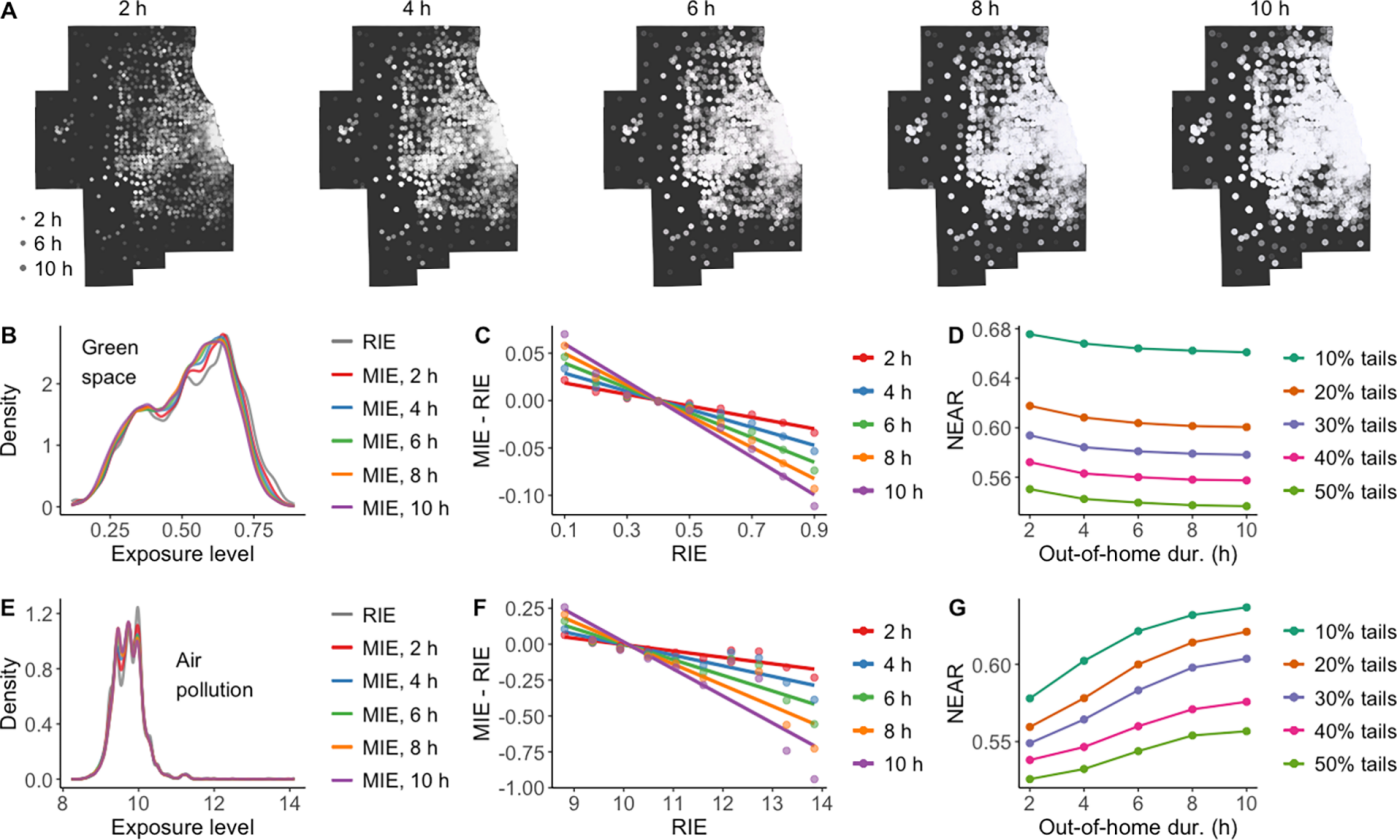
Influence of out-of-home durations on the NEAP. (A) Example simulated mobility data sets with different out-of-home durations in the Chicago Metropolitan Area. (B,E) Comparison of distributions of RIE and MIE to green space (B) and air pollution (E) in simulated data sets with different out-of-home durations. (C,F) Comparison of NEAP in relation to green space (C) and air pollution (F) in simulated data sets with different out-of-home durations, implied by the degree of negative correlation between MIE-RIE and RIE. (D,G) Comparison of the NEAR in relation to green space (D) and air pollution (G) under different out-of-home durations. For each out-of-home duration, the NEAR is measured for the 10%, 20%, 30%, 40%, and 50% tails of the distribution of RIE, and the results are averaged over 999 simulations.

Figures 6A and 7A depict illustrative data sets of human mobility simulations under various travel distances (5 km, 10 km, 15 km, 20 km, 25 km) and out-of-home durations (2 h, 4 h, 6 h, 8 h, 10 h), respectively. Utilizing green space as the environmental variable, it becomes evident that as travel distance increases, the probability distribution of MIE tends to regress toward the RIE’s average value, resulting in a higher likelihood of experiencing the average exposure level (Figure 6B). In Figure 6C, the steepening fitted line with increasing travel distance further confirms the growing prominence of the NEAP in individual exposures. Additionally, Figure 6D also demonstrates that, in scenarios with greater travel distances, an increasing number of individuals with extreme RIE values are likely to experience the NEAP. These findings suggest that longer travel distances of individuals can amplify and promote the NEAP’s effect. This is primarily because greater travel distances increase the probability of experiencing environmental exposures that differ significantly from one’s residential environment, thus magnifying the NEAP’s effect across a larger population.

Similarly, as evident in Figure 7B,C, with increasing out-of-home durations, the trend of neighborhood effect averaging in individual exposures becomes more pronounced. This is because prolonged exposure to the nonresidential environment amplifies the deviation of MIE from RIE. However, the proportion of individuals affected by the NEAP among those with extreme RIE values remains relatively stable (Figure 7D). This is attributed to the relatively static nature of green spaces over time. With unchanged visited locations, an increase in out-of-home duration only augments the weights of nonresidential exposures in MIE, without significantly altering the direction of MIE deviation from RIE. The results suggest that individuals’ longer out-of-home stays enhance, but do not significantly promote, the NEAP in individual exposures to relatively static environmental factors.

For comparison, we further consider a relatively dynamic environmental factor: air pollution. Consistent with the findings in individual green space exposure, the degree and frequency of individual air pollution exposure estimates being influenced by the NEAP increase with greater travel distances (Figure 6F,G). In contrast, when individual travel distances remain constant, an increase in out-of-home duration not only intensifies the extent to which individual air pollution exposure estimates are affected by the NEAP (Figure 7F) but also results in a greater number of individuals experiencing the influence of the NEAP (Figure 7G). This is because the extended time spent outside the home provides more opportunities for exposure to varying levels of air pollution due to its highly dynamic nature over time, thereby promoting the spread of the NEAP’s impact to a larger portion of individuals.

## 4 Discussion

In the course of daily life, individuals are constantly exposed to a diverse array of environments. These daily environmental exposures can further shape individuals’ health and well-being and, in turn, give rise to concerns about environmental and health inequalities.^24,33^ To better characterize individual environmental exposures, we model them as dynamic processes tied to people’s daily mobility, rather than confining them to fixed geographic contexts like residential units. Through this mobility-based approach, our research advances the current comprehension of a fundamental statistical phenomenon in individual environmental exposures, the NEAP, by confirming its universality across various environmental factors and revealing the underlying mechanisms driving its occurrence. The measurement and understanding of the NEAP can statistically quantify and explain the inadequacy and resulting estimation biases that arise from using residence-based units to estimate individual exposures to mobility-dependent environmental factors. This study, on one hand, reveals which social groups and residential locations are more likely to experience such biases in individual exposure estimates caused by the NEAP. On the other hand, it delves deeper into the conditions that contribute to these estimation biases, specifically in terms of environmental variation and human mobility. This, in turn, provides scientific evidence for addressing the NEAP in individual environmental exposure estimates by incorporating human daily mobility, thereby achieving more accurate estimations.

First, using extensive human mobility data in the Chicago Metropolitan Area, we demonstrate the widespread presence of the NEAP in individual mobility-dependent exposures to various environmental factors, including green spaces, air pollution, healthy food environments, transit accessibility, and crime rates. This underscores the critical importance of accounting for human mobility in environmental exposure assessment and its implications for health impacts and inequality research. Second, this study shows that the exposure estimation bias resulting from the NEAP is strongly related to individuals’ socioeconomic attributes and residential locations. For social groups with active daily mobility (e.g., the employed and high-income individuals), their excessively high or low residential exposures are more likely to be mitigated through neighborhood effect averaging compared to those with limited daily mobility (e.g., the unemployed and low-income individuals). Similarly, residents living on the outskirts of urban areas, who have higher travel demands, are more likely to experience the NEAP compared to those residing in the city center. Third, through multiscenario simulations, we uncover the mechanisms behind the NEAP, focusing on two triggering factors: environmental variation and human mobility. The environmental variation within people’s activity spaces is constrained by the inherent spatial autocorrelation of environmental factors, which in turn restricts the degree and frequency of the NEAP’s effect on individual exposure estimates. This indicates that the NEAP is not entirely equivalent to the RTM phenomenon occurring under random variations, but rather its manifestation with the intervention of spatial autocorrelation in environmental factors. Increasing travel distance and out-of-home duration can amplify the NEAP’s impact by widening the gap between MIE and RIE. Additionally, longer travel distances can promote the occurrence of the NEAP among more individuals. However, the promotional effect of out-of-home duration on the NEAP is only significant for highly dynamic environmental factors like air pollution, but not for relatively static ones such as green spaces. These findings underscore, on the one hand, the limitations of conventional residence-based exposure assessment methods, especially when dealing with environmental factors exhibiting significant spatial variation and individuals characterized by active daily mobility. On the other hand, they carry profound implications for the formulation of public health policies and urban planning strategies aimed at enhancing residents’ environmental well-being. For example, increasing transportation subsidies for economically disadvantaged groups to encourage their daily mobility can effectively mitigate prolonged exposure to suboptimal residential neighborhood environments. Furthermore, initiatives like expanding public amenities and employment opportunities on the outskirts of urban areas can facilitate bidirectional travel between urban and suburban regions, thereby neutralizing individuals’ daily exposures.

Our study has several limitations. First, for the sake of computational feasibility, this study aggregates human mobility and environmental data at a 1 km resolution. However, coarser resolution diminishes the ability to capture spatial variation in individual environmental exposures, leading to the stabilization of RIE and MIE differences. This may underestimate or even obscure the impact of the NEAP on exposure estimates, making it difficult to detect meaningful changes in individual exposures during daily mobility. This could further misinform policymakers in formulating environmental and behavioral interventions. Further research is needed to determine effective spatial resolutions and thresholds for identifying the NEAP in mobility-dependent exposures, while also accounting for their potential differences across various environmental factors and regions. Second, this study only discusses the spatially heterogeneous effect of the NEAP. Given the regularity of people’s daily routines, individuals typically exhibit varying mobility levels at different times of the day. Urban environments, such as air pollution, could also follow specific temporal patterns during the day. Consequently, the NEAP may manifest differently at different times, and exert varying degrees of impact on individual exposures. We intend to dedicate more efforts to investigating the temporal effects of the NEAP. Lastly, while we reveal how human mobility contributes to the occurrence of the NEAP, the underlying driving factors remain unclear. In addition to the trade-offs between essential goods and travel costs we discuss here, people’s daily mobility can be influenced by other factors, such as urban structures. Further research efforts will be directed toward understanding how the driving factors behind human mobility impact the occurrence of the NEAP.

## Supporting Information Available

Inference of travel paths; details of environmental data; simulation methods of environmental variation and human mobility; the NEAP in individual environmental exposures; the influence of environmental variation and human mobility on individual exposure estimation bias; data availability (PDF)
