# Supplementary material for: The Universal Neighborhood Effect Averaging in Mobility-Dependent Environmental Exposures
Source: Environ Sci Technol. 2024 Oct 3;58(45):20030–9. doi: 10.1021/acs.est.4c02464 (PMC11562727; doi:10.1021/acs.est.4c02464)
Supplement: Supplementary file 1 — es4c02464_si_001.pdf [file es4c02464_si_001.pdf]

Supporting Information for

The Universal Neighborhood Effect Averaging in Mobility-Dependent Environmental Exposures

Jiannan Cai[a], Mei-Po Kwan[a,b*]

[a]Institute of Space and Earth Information Science, The Chinese University of Hong Kong, Shatin, Hong Kong, China
[b]Department of Geography and Resource Management, The Chinese University of Hong Kong, Shatin, Hong Kong, China

*Mei-Po Kwan (Corresponding Author)
Email: mpk654@gmail.com

This file includes:

Texts S1 to S9
Figures S1 to S8
References

Supporting Information Text

Text S1. Inference of travel paths

Given that the travel survey data lacks the actual movement paths of participants, we deduce each travel path between the origin and destination by assuming that participants selected the route with the shortest travel time corresponding to the reported travel mode (e.g., walking, biking, and driving) for each trip. To obtain realistic travel paths, we retrieve routable networks from OpenStreetMap with different travel modes and then generate the fastest travel paths considering travel modes and the speed limits of different road segments using the OSMnx python package[1]. Finally, we obtain the grid locations where participants were positioned at one-minute intervals by assuming that participants move at a constant speed along each travel path.

Text S2. Environmental data

Green spaces are characterized in this study using the Normalized Difference Vegetation Index (NDVI), which is a widely used measure for quantifying the amount and vigor of vegetation on the land surface using remote sensing. NDVI data, with a resolution of 250 m, are obtained from the online MODIS data repository. We select the data on June 18, 2019, as green features are most prominent during the summer. NDVI values range from -1 to 1, where negative values represent non-biomass such as clouds and water, values close to 0 indicate rocks and bare soil, and higher positive values signify increased greenness. To mitigate biased exposure determination, negative NDVI values are filtered out.

Particulate matter with an aerodynamic diameter $\leq$ 2.5 µm ($PM_{2.5}$) is one of the most significant components of air pollution. We collect hourly $PM_{2.5}$ measurements in the study area from 43 outdoor sensors provided by PurpleAir, a commercial low-cost sensor network. The data is collected on a randomly selected weekday, June 28, 2022, due to the limited availability of outdoor PurpleAir sensors in the study area before that year. To attain more accurate measurements of $PM_{2.5}$, the raw PurpleAir measurements are preprocessed and calibrated against reference-grade $PM_{2.5}$ measurements obtained from the U.S. Environmental Protection Agency (EPA) air quality system (AQS) stations. The calibrated PurpleAir measurements and AQS measurements are then input into an inverse distance weighted interpolation model to estimate hourly $PM_{2.5}$ concentrations at a 1 km resolution in the study area. Details about the preprocessing, calibration, and interpolation of PurpleAir data can be found in Text S3.

To assess the healthy food environment, we collect about 0.5 million points of interest (POIs) that are operational in the Chicago Metropolitan Area between August 2018 and April 2019, sourced from SafeGraph. There POIs are categorized using the North American Industry Classification Codes. Following the classification suggestions provided by the Centers for Disease Control and Prevention[2], we identify 3,268 healthy food retailers and 4,393 less healthy food retailers. Then, the modified retail food environment index (mRFEI)[2] is used to assess the healthy food environment within census tracts, represented as

$$mRFEI = \frac{|HF|}{|HF| + |LF|} \tag{S1}$$

where $|HF|$ and $|LF|$ are the number of healthy food retailers and less healthy food retailers, respectively, within census tracts or 0.5 miles from the tract boundary.

Transit accessibility is measured by the CMAP based on proximity to transit stops. The measurements are calculated using the transit service data in 2017 at the level of Trip Generation zones, also known as subzones. In subzones with transit stops, the proximity measurement for each stop is determined as the average network-based distance to reach that stop without encountering a closer stop. Subsequently, the

proximity measurements to transit stops within each subzone are weighted by the stop's service frequency to calculate the overall proximity measurement for that subzone. For subzones without transit stops, the proximity measurement is the average network-based distance to the five nearest transit stops, weighted by the stop's service frequency. Details about the proximity measurement can be found in ref.[3]. Due to data availability limitations, transit accessibility data only covers seven counties: Cook, DuPage, Kane, Kendall, Lake, McHenry, and Will.

Crime rates, especially those related to violent crimes, are crucial metrics for assessing urban safety. We obtain crime data of the city of Chicago between August 2018 and April 2019 from the Chicago Police Department, which includes crime categories, timestamps, and geographic locations. According to the definition of the National Institute of Justice, we classify murder, robbery, rape, and aggravated assault as violent crimes. Based on 2019 census data, we calculate violent crime rates at the census tract level, expressed as the number of violent crimes per 1,000 residents in each tract.

We aggregate all environmental data into 1 km × 1 km grid cells for easy integration with individual mobility data. Maps for these environmental factors can be found in Figures S4 and S6.

Text S3. Preprocessing, calibration, and interpolation of $PM_{2.5}$ data

A. Preprocessing of PurpleAir $PM_{2.5}$ data. To ensure an adequate number of training samples for our calibration model, we collect hourly $PM_{2.5}$ readings for June 2022 from 113 outdoor PurpleAir sensors located in and around Illinois, U.S., where our study area is situated. To ensure data quality, we first clean the raw PurpleAir $PM_{2.5}$ data following the data preprocessing protocol outlined in ref.[4]. This involves removing outliers based on the dual-channel PurpleAir $PM_{2.5}$ readings, $PM_{2.5}^{A}$ for the Channel A and $PM_{2.5}^{B}$ for the Channel B, using the following steps:

(1) Exclude records with readings from only one channel, as relying solely on one channel's reading makes outlier detection challenging.
(2) Remove records with $PM_{2.5}$ readings exceeding 3,000μg/m^3 in both channels, indicating apparent outliers.
(3) Calculate the absolute percentage bias (APB) of dual-channel readings (eq S2), and exclude records with the top 5% largest APB.

$$\mathrm{APB(A,B)} = \left| \frac{\mathrm{PM}_{2.5}^{B} - \mathrm{PM}_{2.5}^{A}}{\mathrm{PM}_{2.5}^{A}} \right| \tag{S2}$$

Figure S1 compares PurpleAir dual-channel $PM_{2.5}$ readings before and after removing outliers with the top 5% largest bias. Initially, the records display a substantial dual-channel discrepancy with an R^2 of 0.27 and a slope of 0.97. After removal, the remaining data demonstrate a notably improved dual-channel agreement with an R^2 of 0.96 and a slope of 1. Finally, the cleaned dual-channel readings are averaged to obtain the $PM_{2.5}$ measurement for each PurpleAir sensor.

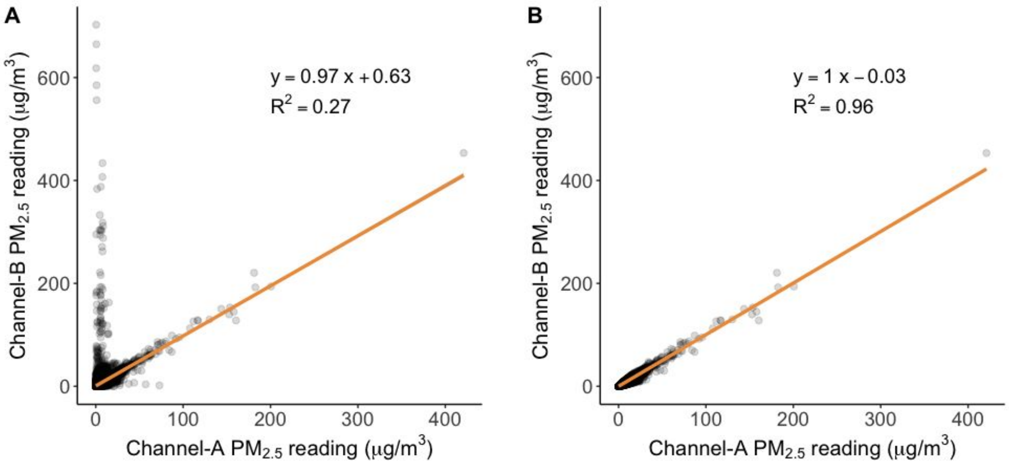

Figure S1. Scatter plots of PurpleAir dual-channel hourly $PM_{2.5}$ readings (A) before and (B) after removing outliers with the top 5% largest absolute percentage bias.

B. Calibration of PurpleAir $PM_{2.5}$ data. To attain more accurate measurements of $PM_{2.5}$, we further calibrate the PurpleAir measurements against the "gold-standard" hourly measurements obtained from the U.S. Environmental Protection Agency (EPA) air quality system (AQS) stations. However, there are significant spatial disparities in the placement of PurpleAir sensors and AQS stations in Illinois, to the extent that no PurpleAir/AQS pair can be found within the same 1 km × 1 km grid cell. Therefore, we expand the search radius, pairing PurpleAir sensors and AQS stations within a 10 km range, and obtain a total of 19 PurpleAir/AQS pairs (see Figure S2A). To ensure comparability between PurpleAir and AQS $PM_{2.5}$ measurements, we conduct inverse distance weighted (IDW) interpolation based on hourly $PM_{2.5}$ measurements from all AQS stations to estimate $PM_{2.5}$ concentrations at locations of paired PurpleAir sensors. Then, these AQS-based IDW estimates serve as benchmarks for calibrating $PM_{2.5}$ measurements from paired PurpleAir sensors. In practice, some PurpleAir sensors are installed in residential areas, and their readings may be influenced by factors such as cigarette smoke, barbecues, fireplaces, and traffic, leading to high-level local pollution in microenvironments[4, 5]. To mitigate the impact of high-level local pollution in the microenvironments of PurpleAir sensors, we calculate the APB of paired PurpleAir/AQS $PM_{2.5}$ measurements (eq S3), $PM_{2.5}^{PurpleAir}$ and $PM_{2.5}^{AQS}$, and exclude outliers with the top 5% largest APB.

$$\mathrm{APB(AQS, PurpleAir)} = \left| \frac{PM_{2.5}^{PurpleAir} - PM_{2.5}^{AQS}}{PM_{2.5}^{AQS}} \right| \qquad \text{(S3)}$$

This process yields a total of 9,769 paired hourly $PM_{2.5}$ measurements (see Figure S2B).

A linear regression analysis of uncalibrated PurpleAir measurements against AQS-based IDW estimates results in an R^2 of 0.13. This suggests the presence of an underlying nonlinear relationship between the paired PurpleAir/AQS hourly $PM_{2.5}$ measurements, posing a challenge to fitting them with a predefined distribution model. Thus, we employ a well-established distribution-free machine learning model, random forest[6], to calibrate the PurpleAir measurements, using AQS estimations as the response variable. In addition, recognizing the influence of temperature, relative humidity, sensor operating time, and sensor uptime on the data quality of low-cost sensors[4], we include these four parameters provided by PurpleAir sensors as covariates in our calibration model.

To validate our calibration model, we randomly split the paired PurpleAir/AQS dataset into a training set (80%) for model training and a test set (20%) for model assessment. For the training set, we also execute

10-fold cross-validations (CV). The CV R^2 and root-mean-square error (RMSE) are 0.83 and 2.2, respectively, indicating high model accuracy and consistency in calibrating PM2.5 measurements from PurpleAir sensors. Figure S3 shows scatter plots of paired PurpleAir/AQS $PM_{2.5}$ measurements before and after calibration on the remaining test set. One can see that the uncalibrated PurpleAir $PM_{2.5}$ measurements exhibit substantial discrepancies compared to AQS estimations, with an R^2 of 0.12 and a RMSE of 7.88. After calibration, the agreement between paired PurpleAir and AQS measurements is significantly enhanced, yielding an elevated R^2 of 0.7 and a reduced RMSE of 3.49. The results demonstrate that our random forest calibration model can significantly improve the accuracy of PurpleAir $PM_{2.5}$ measurements and can be effectively generalized to previously unseen samples.

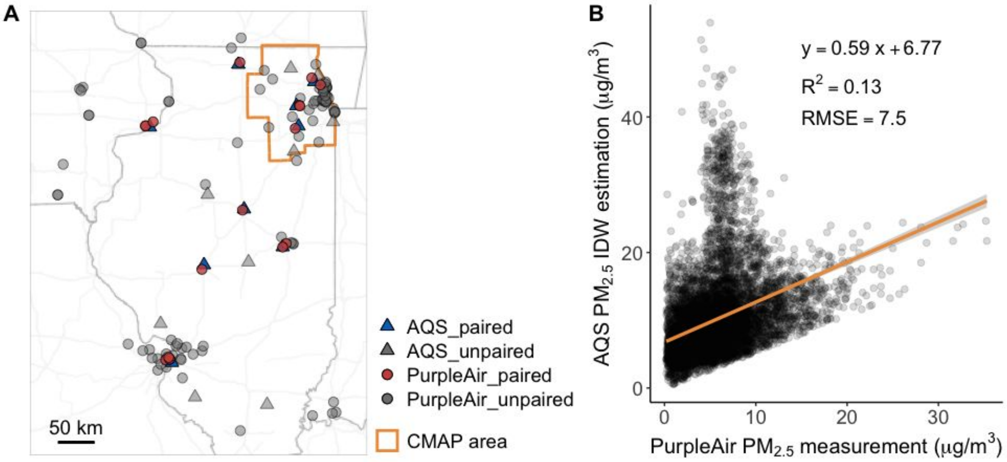

Figure S2. Pairing of PurpleAir and AQS sites and $PM_{2.5}$ measurements. (A) Spatial distribution of PurpleAir sensors and AQS stations. PurpleAir/AQS pairs are highlighted in red (PurpleAir) and blue (AQS). (B) Paired PurpleAir/AQS $PM_{2.5}$ measurements. AQS measurements are AQS-based IDW estimates for $PM_{2.5}$ concentrations at locations of paired PurpleAir sensors.

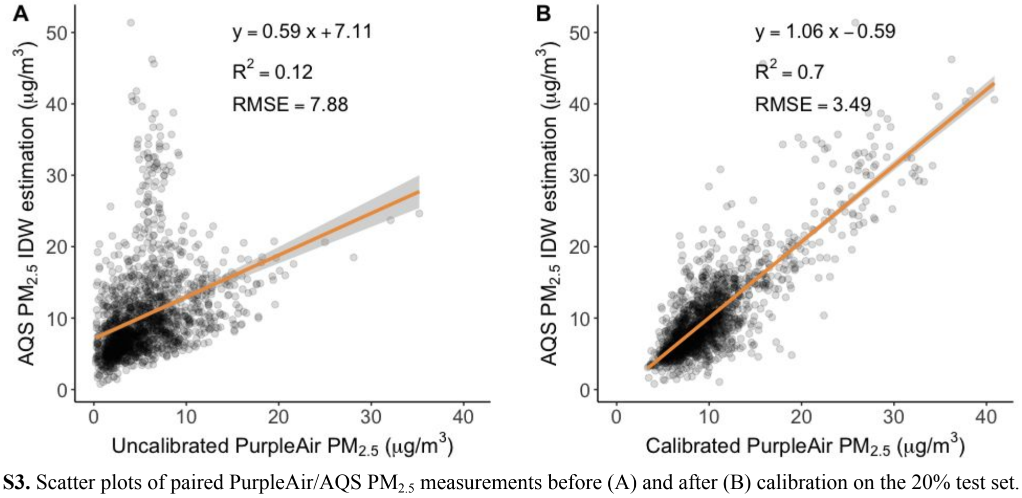

Figure S3. Scatter plots of paired PurpleAir/AQS $PM_{2.5}$ measurements before (A) and after (B) calibration on the 20% test set.

C. Interpolation of $PM_{2.5}$ data. We extract hourly data from 43 outdoor PurpleAir sensors and 7 AQS stations in the Chicago Metropolitan Area on a representative workday, June 28, 2022, to model the variations in $PM_{2.5}$ concentrations throughout the day. For PurpleAir sensors paired with AQS stations, we replace their $PM_{2.5}$ measurements with IDW estimates based on AQS data to better represent the pollution levels. For those without a paired AQS station, we correct their $PM_{2.5}$ measurements using our trained random forest calibration model. Then, AQS $PM_{2.5}$ measurements and calibrated PurpleAir PM2.5 measurements are jointly

input into a IDW interpolation model to generate hourly 1 km-resolution gridded estimates of $PM_{2.5}$ concentrations within the study area.

As shown in Figure S4A, there are noticeable variations in the spatial distribution of $PM_{2.5}$ concentrations throughout the day. In general, $PM_{2.5}$ pollution is most severe around midday, while air quality tends to be better in the early morning and evening. This pattern could be attributed to factors such as increased vehicular and industrial emissions during peak daytime hours, coupled with atmospheric conditions that are less conducive to pollutant dispersion.

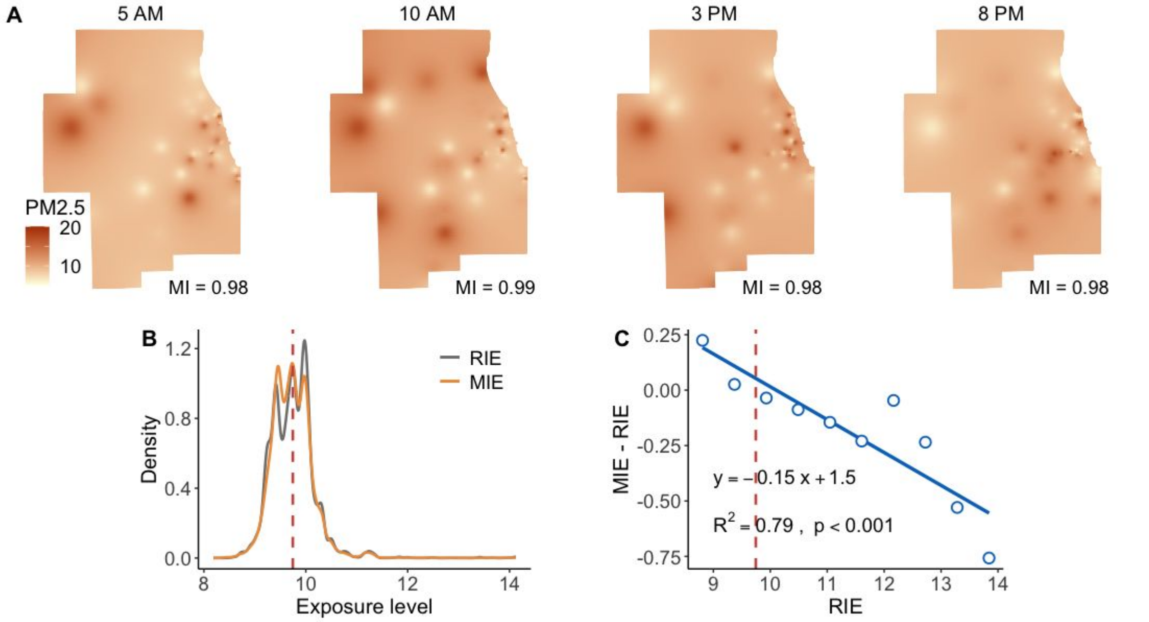

Figure S4. Air pollution exposure. (A) Spatial distribution of estimated hourly $PM_{2.5}$ concentrations in the study area at different times (5 AM, 10 AM, 3 PM and 8 PM). (B) Comparison of distributions of RIE and MIE to $PM_{2.5}$ pollution. (C) Evidence of the NEAP in individual air pollution exposure. The red vertical dashed line represents the average value of RIE.

Text S4. Environmental variation simulation

We develop an environmental variation simulation method to obtain simulated environmental datasets with desired environmental variation levels. For each environmental scenario, we characterize environmental variation using a well-established spatial autocorrelation statistic, Moran's *I* (MI)[7]. A higher MI value indicates a stronger spatial autocorrelation of the environmental factor within the study area, signifying lower environmental variation. To achieve a targeted MI value, we employ the distribution-free simulation strategy in the spatial autocorrelation reconstruction method[8] to generate stochastic replicates of an observed environmental dataset conditioning on the desired environmental variation level.

The method begins by randomly permuting the attributes of the observed environmental dataset (e.g., NDVI) across spatial locations (1 km × 1 km grid cells) within the study area. Subsequently, the permuted dataset is iteratively modified by swapping the environmental attributes at two randomly selected spatial locations. The objective is to minimize the disparity between the MI value of the current permuted dataset, MI_i, and the targeted MI value, MI_t, represented by

$$\Delta MI_i = |MI_i - MI_t| \tag{S4}$$

The ith permuted dataset is accepted if its environmental variation level, in comparison to that of the previously accepted (i-1)th permuted dataset, is closer to the targeted level, i.e., $\Delta MI_i < \Delta MI_{i-1}$. The modification process terminates when the ΔMI_i becomes smaller than a tiny threshold (0.01 in this study) or when the number of iterations exceeds a specified limit (100,000 in this study).

Text S5. Human Mobility Simulation

We utilize two characteristics, daily travel distance and out-of-home duration, to measure the level of human mobility, and develop corresponding simulation methods to generate simulated human mobility datasets with varying mobility levels. The simulation of human mobility is conducted over 1 km × 1 km grid cells. For each individual, we simulate the grid cells he or she visits and traverses throughout the day, as well as the duration in each grid cell.

In the simulation with the travel distance constraint, we maintain the individuals' home locations and out-of-home durations consistent with those in the observed dataset. To constrain travel distances to a desired level, we employ a Poisson distribution with a mean value of μ to generate the travel distance for each individual. At the generated distance from each individual's home location, we randomly select a spatial location within the study area as his or her out-of-home location, under the assumption that each individual visits only one location outside his or her home. Then, we simulate the travel path using a spatial random walk model. It begins at each individual's home location and iteratively chooses one of the neighboring grid cells at random to approach the destination. This process results in a sequence of random steps connecting each individual's home location to the out-of-home location. After obtaining the travel path of each individual, we estimate the travel time based on the distance-speed relationship learned from the observed dataset (see Text S6), and evenly allocate it to each grid cell along the path. Furthermore, we match each individual's duration at the out-of-home location to the observed value, while allocating any remaining time in the day to the duration at the home location.

When simulating human mobility with regard to out-of-home duration, we keep each individual's home location, out-of-home locations, travel paths, and travel times consistent with those in the observed dataset. To achieve the desired out-of-home duration, we employ a Poisson distribution with a mean value of τ to generate the total out-of-home duration for each individual and allocate it to different out-of-home locations based on observed proportions. Any remaining time is then allocated to the duration at the home location.

Text S6. Travel time estimation in simulated scenarios of human mobility

In the simulation of human mobility scenarios with different travel distances, we estimate the travel time of each simulated travel path based on the travel distance-speed relationship learned from the observed human mobility dataset. Specifically, for every journey in the observed dataset, we compute the associated travel distance and speed. Then, employing 5km intervals, we derive the median travel speed for each range of travel distances, ensuring a more robust estimation of average speed. As shown in Figure S5, there exists significant variability in travel speeds across different distance intervals. This is primarily due to people having varied travel mode preferences depending on the travel distances. For instance, when the travel distance is less than 5km, 21.3% of journeys involve walking, with the proportion significantly decreasing as the distance increases. Consequently, we observe the minimum travel speeds for distances less than 5km. Following this observed pattern, we query the median travel speed within the distance interval corresponding to each simulated travel patch, and then achieve a reasonable estimation of travel time in simulated scenarios.

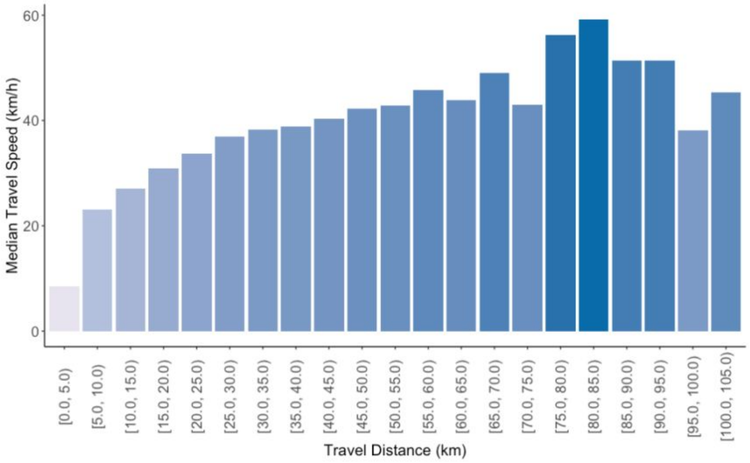

Figure S5. Average travel speeds for different travel distances in the observed human mobility dataset.

Text S7. The NEAP in individual exposures to different environmental factors

We evaluate mobility-based and residence-based individual exposures (MIE and RIE), respectively, to different environmental factors, including green spaces, air pollution, healthy food environment, transit accessibility, and crime rates. As shown in Figures S4B and S6B,E,H,K, for a given environmental factor, the distribution patterns of mobility-based and residence-based individual exposures (MIE and RIE) are generally similar. However, they do not necessarily follow the bell-shaped curve that was empirically observed in previous studies[9–11]. The diverse distribution patterns of individual environmental exposures can be attributed to the differences in the degree of interaction between human activities and environmental factors, caused by the distinct spatiotemporal dynamics of the environmental factors. Despite this, the MIE typically shows a relatively higher probability at the average exposure level of all participants (see the red vertical dashed line). This suggests that when considering individual mobility patterns, people are more likely to experience the average level of environmental exposures.

Using the RIE as a baseline, we further assess the average variation of MIE (i.e., MIE − RIE) across different RIE intervals. Figure S4C and S6C,F,I,L provide strong statistical evidence for the universality of the neighborhood effect averaging problem (NEAP) in individual exposures to various environmental factors. We demonstrate a consistent and significant negative correlation between MIE-RIE and RIE across different environmental factors. Specifically, the linear regressions show slopes ranging from -0.19 to -0.15, with R^2 values from 0.79 to 0.97 and p-values all less than 0.001. Additionally, around the average value of RIE, the direction of variation in MIE tends to change. This implies that individuals residing in areas with environmental exposure levels below (or above) the average tend to experience higher (or lower) exposure levels during their daily mobility. The results corroborate the patterns of upward averaging and downward averaging of individual environmental exposures when considering people's daily mobility.

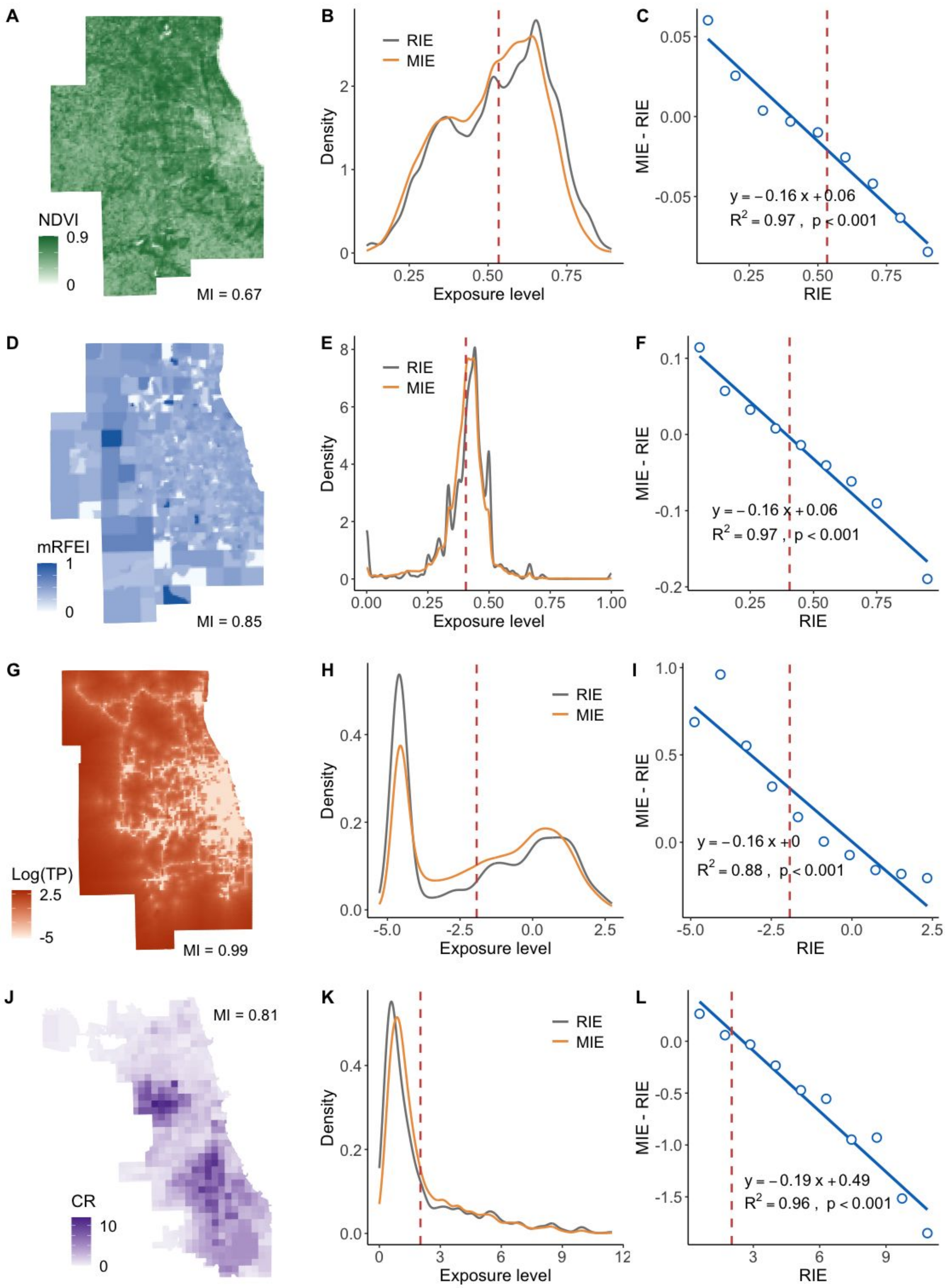

Figure S6. Spatial distribution of (A) green spaces, (D) healthy food environment, (G) transit accessibility, and (J) crime rates. Comparison of distributions of RIE and MIE to (B) green spaces, (E) healthy food environment, (H) transit accessibility, and (K) crime rates. The red vertical dashed line represents the average value of RIE. (C) Evidence of the NEAP in individual exposures to (C) green spaces, (F) healthy food environment, (I) transit accessibility, and (L) crime rates. The red vertical dashed line represents the average value of RIE. The values of transit proximity are logarithmically transformed for better interpretation and visualization.

Further, using green space exposure as an example, we perform sensitivity testing to assess the impact of resolution choice on the statistical evidence for the NEAP. Figure S7 shows that at different finer resolutions (250 m, 500 m, and 750 m), the variation of MIE with changes in RIE is highly consistent in both direction and magnitude with the result obtained at a 1 km resolution (Figure S6C). Specifically, the slopes of the linear regressions are -0.16 or -0.17, with R^2 values of 0.97 or 0.98, and *p*-values all below 0.001. This consistency implies that the NEAP manifests in individual green space exposure in a similar form and degree across all tested resolutions. Therefore, the grid resolution of 1 km used in this study is effective for the empirical verification of the NEAP.

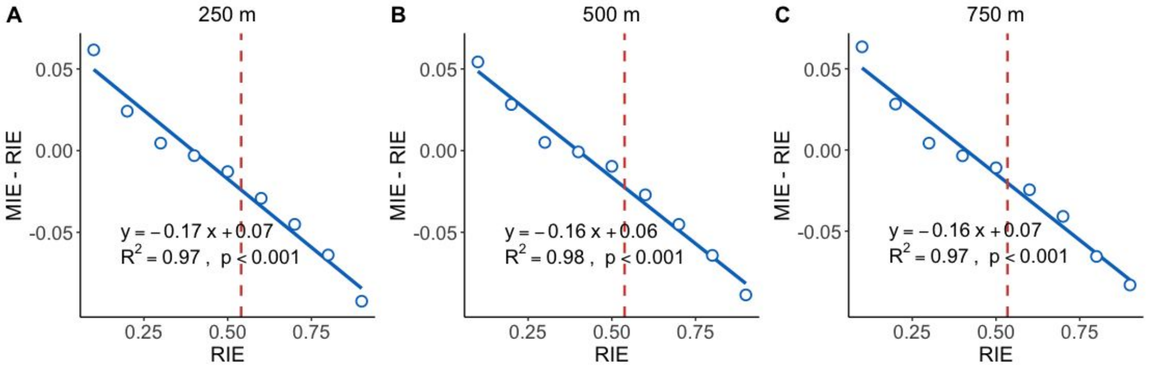

Figure S7. Evidence of the NEAP in individual exposures to green spaces obtained from data with (A) 250m, (B) 500m, and (C) 750m resolutions. The red vertical dashed line represents the average value of RIE.

Text S8. The influence of environmental variation and human mobility on individual exposure estimation bias

Using green space as the environmental variable, Figure S8 illustrates how individual exposure estimation bias is influenced by environmental variation and human mobility. When considering each influencing factor individually, a non-linear descriptive relationship is observed between individual exposure estimation bias and the environmental variation within an individual's activity space, daily travel distance, or duration of stay at out-of-home locations (Figure S8A–C). As one of these factors increases, the bias initially shows an increase, followed by a decrease. Figure S8D–F further reveals that the variation of one influencing factor may also be accompanied by changes in the other two factors. Thus, to better understand the individual exposure estimation bias caused by the NEAP, it is essential to consider the joint effects of these environmental variations and human mobility factors. When considering individuals within the same interval of travel distance, the bias shows a clear increase with the rise of environmental variation experienced in their daily activity space (Figure S8D). Similar trends can also be observed between bias and the other two pairs of influencing factors (Figure S8E,F). By controlling for more individual socio-demographic and residential location factors, the spatial error model further demonstrates significant positive correlations between estimation bias and environmental variation within the activity space, daily travel distance, and duration of stay at out-of-home locations (Figure 3E).

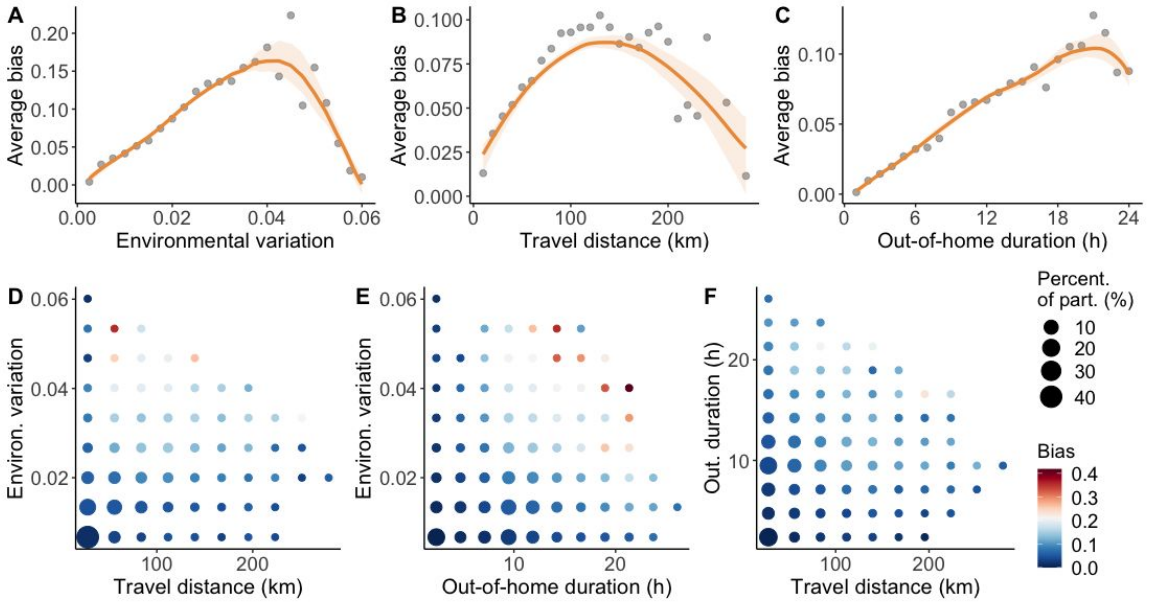

Figure S8. How do environmental variation and human mobility affect the bias in estimating individual exposure? (A–C) Average bias with varying environmental variation (measured as the variance of Normalized Difference Vegetation Index (NDVI) values) within the daily activity space of each individual (A), varying total travel distance (B), and varying duration of stay at out-of-home locations (C). Each point represents the average estimation bias within a specific interval of the corresponding variable. The curve is the non-parametric fit based on local polynomial kernel smoothers. The shaded area is the 95% confidence interval of each fitting curve obtained using the bootstrap method. (D–F) Average bias with varying environmental variation and travel distance (D), varying environmental variation and out-of-home duration (E) and varying travel distance and out-of-home duration (F). Colors correspond to the average estimation bias and the size is proportional to the percentage of participants within specific intervals of the two corresponding variables.

Text S9. Data availability statement

Travel survey data and transit proximity data are publicly available on the CMAP Data Hub (https://datahub.cmap.illinois.gov). NDVI data are available for download from the online MODIS data repository (https://modis.gsfc.nasa.gov). PurpleAir $PM_{2.5}$ data can be accessed through the PurpleAir API (https://api.purpleair.com). EPA AQS $PM_{2.5}$ data are publicly available on the EPA website (https://aqs.epa.gov/aqsweb/airdata/download_files.html). Crime data are available on the Chicago Data Portal (https://data.cityofchicago.org). Census data are available online (https://www.census.gov). SafeGraph POI data are commercially available and may be requested for research use (https://www.safegraph.com/contact-us).

References

1. Boeing, G. OSMnx: New methods for acquiring, constructing, analyzing, and visualizing complex street networks. *Comput. Environ. Urban Syst.* **2017**, *65*, 126–139.
2. CDC (Centers for Disease Control and Prevention). *Children's Food Environment State Indicator Report*, 2011. https://www.cdc.gov/obesity/downloads/ChildrensFoodEnvironment.pdf (accessed May 12, 2023).
3. CMAP (Chicago Metropolitan Agency for Planning). *Go To 2040 Update Appendix: Indicator Methodology*, 2015). https://www.cmap.illinois.gov/documents/10180/332742/Update+Indicator+Methodology+FINAL.pdf (accessed May 20, 2023).
4. Bi, J.; Wildani, A.; Chang, H. H.; Liu, Y. Incorporating low-cost sensor measurements into high-

resolution pm2.5 modeling at a large spatial scale. *Environ. Sci. Technol.* **2020**, *54* (4), 2152–2162.

5. Zheng, T.; Bergin, M. H.; Johnson, K. K.; Tripathi, S. N.; Shirodkar, S.; Landis, M. S.; Sutaria, R.; Carlson, D. E. Field evaluation of low-cost particulate matter sensors in high-and low-concentration environments. *Atmospheric Measurement Techniques* **2018**, *11* (8), 4823–4846.
6. Breiman, L. Random Forests. *Mach. Learn.* **2001**, *45* (1), 5–32.
7. Moran, P. A. P. Notes on continuous stochastic phenomena. *Biometrika* **1950**, *37* (1-2), 17–23.
8. Cai, J.; Kwan, M.-P. Detecting spatial flow outliers in the presence of spatial autocorrelation. *Comput. Environ. Urban Syst.* **2022**, *96*, 101833.
9. Dewulf, B.; Neutens, T.; Lefebvre, W.; Seynaeve, G.; Vanpoucke, C.; Beckx, C.; Van de Weghe, N. Dynamic assessment of exposure to air pollution using mobile phone data. *Int. J. Health Geogr.* **2016**, *15*, 14.
10. Nyhan, M. M.; Kloog, I.; Britter, R.; Ratti, C.; Koutrakis, P. Quantifying population exposure to air pollution using individual mobility patterns inferred from mobile phone data. *J. Expo. Sci. Environ. Epidemiol.* **2019**, *29* (2), 238–247.
11. Kim, J.; Kwan, M.-P. How neighborhood effect averaging might affect assessment of individual exposures to air pollution: A study of ozone exposures in Los Angeles. *Ann. Assoc. Am. Geogr.* **2021**, *111* (1), 121–140.
